# Bringing Precision to Pediatric Care: Explainable AI in Predicting No-Show Trends Before and During the COVID-19 Pandemic

**DOI:** 10.3390/bioengineering12030227

**Published:** 2025-02-24

**Authors:** Quincy A. Hathaway, Naveena Yanamala, TaraChandra Narumanchi, Janani Narumanchi

**Affiliations:** 1 Aspirations LLC, Morgantown, WV 26505, USA; quincy.hathaway@pennmedicine.upenn.edu (Q.A.H.); tarachandra_m_narumanchi_md@hotmail.com (T.N.); 2 Department of Radiology, University of Pennsylvania, Philadelphia, PA 19104, USA; 3 Rutgers Robert Wood Johnson Medical School, Rutgers University, New Brunswick, NJ 08901, USA; yanamala.naveena@rutgers.edu; 4 Chicagi LLC, Ste R 10 N Gould Street, Sheridan, WY 82801, USA; 5 Le Bonheur Children’s Hospital, University of Tennessee, Memphis, TN 38103, USA

**Keywords:** patient no-shows, machine learning, predictive modeling, XGBoost, explainable AI

## Abstract

Patient no-shows significantly disrupt pediatric healthcare delivery, highlighting the necessity for precise predictive models, especially during the dynamic shifts caused by the SARS-CoV-2 pandemic. In outpatient settings, these no-shows result in medical resource underutilization, increased healthcare costs, reduced access to care, and decreased clinic efficiency and increased provider workload. The objective is to develop a predictive model for patient no-shows using data-driven techniques. We analyzed five years of historical data retrieved from both a scheduling system and electronic health records from a general pediatrics clinic within the WVU Health systems. This dataset includes 209,408 visits from 2015 to 2018, 82,925 visits in 2019, and 58,820 visits in 2020, spanning both the pre-pandemic and pandemic periods. The data include variables such as patient demographics, appointment details, timing, hospital characteristics, appointment types, and environmental factors. Our XGBoost model demonstrated robust predictive capabilities, notably outperforming traditional “no-show rate” metrics. Precision and recall metrics for all features were 0.82 and 0.88, respectively. Receiver Operator Characteristic (ROC) analysis yielded AUCs of 0.90 for all features and 0.88 for the top five predictors when evaluated on the 2019 cohort. Furthermore, model generalization across racial/ethnic groups was also observed. Evaluation on 2020 telehealth data reaffirmed model efficacy (AUC: 0.90), with consistent top predictive features. Our study presents a sophisticated predictive model for pediatric no-show rates, offering insights into nuanced factors influencing attendance behavior. The model’s adaptability to evolving healthcare delivery models, including telehealth, underscores its potential for enhancing clinical practice and resource allocation.

## 1. Introduction

Patient no-shows, defined as a patient failing to attend a scheduled appointment without prior notification, vary widely across healthcare settings, ranging from 12% to 80% [1]. No-shows are a major logistical and economic challenge for clinics and hospital systems, leading to significant revenue losses. No-shows cost the United States healthcare system over USD 150 billion annually, with individual physicians losing an average of USD 200 per unused time slot [2]. Whether patients show up or not, healthcare organizations and medical practices must still pay their staff and cover expenses of resources/facilities. For the provider, no-showed appointments decrease the volume of medical care that can be given. For patients, the increasing lengths of time needed to schedule follow-up appointments can be prohibitive of receiving proper care [3]. Patients who missed an appointment were up to 70% less likely to return within 18 months, particularly older patients with chronic illnesses who often discontinued care after a single missed visit [4].

Several studies have modeled strategies for reducing patient wait times for clinic appointments through artificial intelligence (AI)-enabled technologies and solutions [5]. These machine and deep learning approaches have utilized random forest [6,7], logistic regression [6,8], gradient boosting [9,10], ensemble-based models [11,12], deep neural networks [13,14], and various other approaches to predict patient no-shows. Studies have also examined the type of visit, whether primary care or specialty clinic, and varying demographic populations (e.g., United States, Saudi Arabia, Brazil, etc.) [5]. While outpatient scheduling with a healthcare provider is traditionally thought to account for the largest percentage of no-showed appointments, the use of no-show algorithms could also predict patient readmissions [15] and attrition from diagnostic visits (i.e., imaging and laboratory studies) [16]. These individualized approaches boost patient/provider satisfaction [17] and allow for a more efficient model of healthcare.

The factors affecting patient no-shows can vary slightly between studied populations but can be generalized into three major categories: (1) modifiable and unmodifiable patient characteristics (e.g., age, sex, race/ethnicity, BMI), (2) type of appointment scheduled (i.e., primary care or specialty clinic), and (3) patient behavior (e.g., previous no-show rate, time-to-appointment, travel distance, weather, etc.) [5]. Previous research indicates patients who miss appointments tend to be of lower socioeconomic status, and often have a history of failed/no-show appointments, government-provided health benefits, and psychosocial problems and are less likely to understand the purpose of the appointment [1,9,18]. In addition to forgetting appointments, issues such as trouble getting off work, trouble finding childcare, transportation, and cost can also limit patient compliance for an appointment. No-show rates also increase with increasing time between scheduling and the actual appointment [19]. In pediatrics, few studies have examined how no-show rates can be predicted using machine learning [20,21], with no pediatric studies exploring how the SARS-CoV-2 pandemic affects the ability of no-show rates to handle virtual appointments.

Our study assesses the ability of a machine learning model to predict pediatric no-show rates before and during the SARS-CoV-2 pandemic. We utilized electronic medical record (EMR) data for patients, including features related to modifiable and unmodifiable patient characteristics, appointment type, and patient behavior. We built our model on pre-pandemic outpatient appointment data and utilized pre-pandemic- and pandemic-derived external validation sets. We were able to effectively predict pediatric no-show rates in our validation sets and further explored the role of race/ethnicity in no-show rate prediction.

## 2. Methods

### 2.1. Ethical Approval

The study was conducted in accordance with the Declaration of Helsinki and approved by the Institutional Review Board of West Virginia University (protocol number: 2002907160, date of approval: 17 March 2020) for studies involving humans.

### 2.2. Study Population and Data Preprocessing

Data from medical appointments were gathered from an outpatient clinic within a prominent academic pediatric hospital (West Virginia University Hospitals), aimed at enhancing quality improvement efforts (Table 1). The dataset includes appointments from all clinic departments, covering both main campus and satellite locations, as well as various visit types. It also includes healthcare provider details and environmental factors such as weather conditions. To facilitate analysis, categorical data were transformed into multiple binary variables. For instance, the original “day of the week of the appointment” feature, ranging from 1 to 7 (representing Sunday to Saturday), was converted into seven binary indicators, each indicating the appointment’s occurrence on a specific day. Numerical features were normalized to a range of 0 to 1. The labels indicating appointment outcomes were binary, with 1 indicating a no-show and 0 denoting attendance. Notably, a single patient may have multiple records due to multiple appointments. The patient demographic largely comprises children, often accompanied by their parents or caregivers to appointments.

### 2.3. Machine Learning Algorithm Development

Our current model leverages pediatric appointments from 2015–2018 (training/testing), 2019 (validation), and 2020 (external holdout) that were scheduled at West Virginia University Outpatient Clinics under the West Virginia University Hospital Systems in West Virginia. The datasets consist of 209,408 (2015–2018), 82,925 (2019), and 58,820 (2020) patient appointments. A total of 46 features were collected that included demographic factors, time of appointment, hospital variables, type of appointment scheduled, and environmental conditions.

Our machine learning model relies on a XGBoost framework that allows for adaptable weighting of variables and hyperparameter optimization [22]. To predict patient no-shows, we implemented the XGBoost algorithm using the XGBClassifier, chosen for its robust performance on complex datasets. The classifier was configured with a gradient boosting “gbtree” booster and a base score of 0.5. We set the learning rate to 0.3 and the maximum depth of trees to 6, ensuring the model was sufficiently detailed yet avoided overfitting. Each tree node considered all features due to a subsample rate and colsample parameters set to 1. The model utilized 100 estimators, with the optimization objective set to binary logistic, tailored for binary classification tasks.

For regularization, we applied L2 regularization (lambda = 1) and L1 regularization (alpha = 0) to balance model complexity and performance. The model automatically handled missing values by treating them as NaNs, ensuring flexibility in dealing with incomplete records. The model operated under a binary logistic objective, focusing on the probability of no-show events. A total of four parallel jobs were run (*n_jobs* set to 4), exploiting multi-core processing to expedite computation. The random state was anchored at 0 to ensure consistency and reproducibility across model runs. We employed an automatic predictor setting, which optimally selected the most efficient prediction method based on the data structure. The tuning of hyperparameters like *max_depth, min_child_weight, subsample, and colsample_bytree* was conducted through a methodical grid search to identify the optimal balance, enhancing model effectiveness without overfitting. This methodological approach was geared towards developing a robust predictive model that could effectively forecast patient no-show probabilities in pediatric outpatient settings, considering various patient and environmental factors.

### 2.4. Performance Evaluation Metrics

The model’s performance was evaluated using standard metrics: accuracy, precision, recall, and area under the ROC curve (AUROC). The ROC curve plots the true positive rate (sensitivity) against the false positive rate (1-specificity) at different thresholds ranging from 0 to 1. The prediction scores (i.e., the predicted probabilities of no-shows) are compared at each threshold. A higher AUROC value (closer to 1) indicates better prediction quality. Similarly, the precision–recall curve (PRC) and area under the PRC (AUPRC) were calculated. Precision measures the accuracy of positive predictions, while recall measures the model’s ability to identify all actual positives. These metrics collectively provide insights into the model’s predictive accuracy and effectiveness.

### 2.5. SHAP Feature Analysis

We employed SHAP (SHapley Additive exPlanations) feature analysis to interpret the predictive model’s behavior and understand the importance of each feature in making predictions. SHAP values provide insights into how individual features contribute to the model’s output. By decomposing the model’s output for each prediction, SHAP enables us to understand the impact of each feature on the prediction outcome. In particular, SHAP summary plots are generated to visualize the overall feature importance and understand the relationship between specific features and the predicted outcome. Positive SHAP values indicate a feature that contributes to increasing the prediction, while negative values suggest a feature that decreases the prediction.

### 2.6. Intellectual Property/Data Availability

Our machine learning algorithm is covered by a provisional patent filed between Aspirations LLC and West Virginia University. This is distinctly unique from other filed patents, including the following: US0150242819A1 [2015]—utilizing advanced statistical techniques with no indication of accuracy or performance of the models. US20110208674A1 [2010]—a similar concept but within a ticket booking system. WO2018058189A1 [2016]—describes a supervised learning module that targets overbooking strategies, rather than uniquely identifying patients who are at risk of no-showing their appointment. Due to confidentiality agreements and the proprietary nature of the technology, the underlying data supporting this work are not publicly available.

### 2.7. Statistics

Baseline characteristics of the dataset were analyzed to provide insights into the demographic, clinic-based, insurance, and appointment-related attributes of the patient population. Descriptive statistics, including measures of central tendency and dispersion, were computed for numerical variables such as age and appointment duration, etc. Categorical variables, such as gender and appointment type, were summarized using frequency distributions.

To identify factors influencing the likelihood of patient no-shows, univariate and multivariate analyses were conducted. Univariate analyses involved assessing the association between each individual predictor variable and the outcome variable (completed vs. show) using appropriate statistical tests such as chi-square tests for categorical variables and *t*-tests or ANOVA for continuous variables.

## 3. Results

### 3.1. Baseline Characteristics and Influence of Variables on No-Show Rates

**Figure 1:**
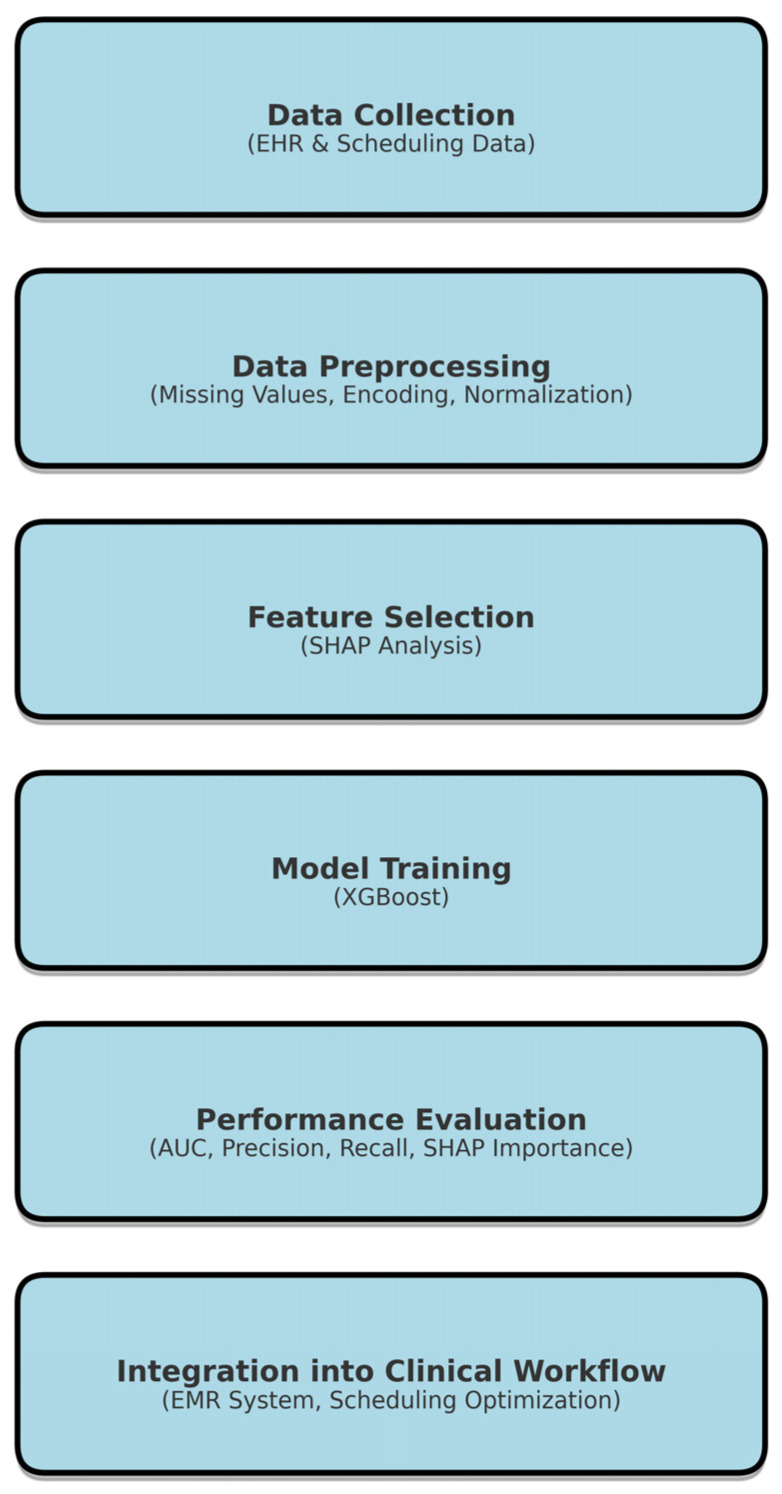
Research framework for predicting patient no-shows using XGBoost. Sequential steps, including data collection from electronic health records (EHR), preprocessing techniques, feature selection using SHAP analysis, model training with XGBoost, performance evaluation using AUC and AUC metrics, and integration into clinical workflows for scheduling optimization. The structured process ensures efficient and interpretable predictive modeling for healthcare applications.

We retrospectively collected records from 161,822 hospital appointments made by 19,450 patients between 1 January 2015 and 31 December 2019 at pediatric clinics of all specialties in West Virginia University Hospitals [WVUH]. Figure 1 describes the workflow for the project. From our experience, the main factors driving the no-show rate were the days until the scheduled appointments. The longer the interval, the lower the likelihood of the appointment being completed. We also noted that same day appointments had low likelihood of patient no-shows. Appointment cancellations or reschedules were also strong predictors of no-shows. Interestingly, full-time employment status of the parent had a positive impact on adherence to the appointment in our pediatric clinics. Those with previous history of no-shows tended to have more chances of missing future appointments. Although not a major factor, some pediatric specialties exhibited higher appointment adherence rates, for example cardiology and nephrology, while others had higher chances of experiencing patient no-shows, for example hematology, neurology, and gastroenterology.

Gender, weather, race or ethnicity, and language preference did not significantly impact the no-show status for a scheduled appointment.

### 3.2. Performance of Model Predictions of No-Shows

**Figure 2:**
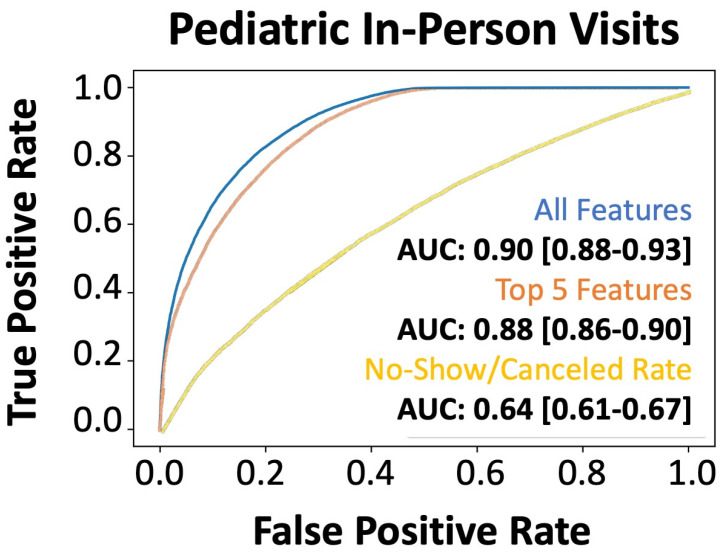
Performance of the machine learning (XGBoost) model for predicting no-shows in the holdout test set. AUROC of xgboost machine learning model: all features (blue line), only the top five features (orange line), and the direct no-show/cancel rate (yellow line) in predicting no-shows for the 2019 holdout validation dataset.

**Figure 3:**
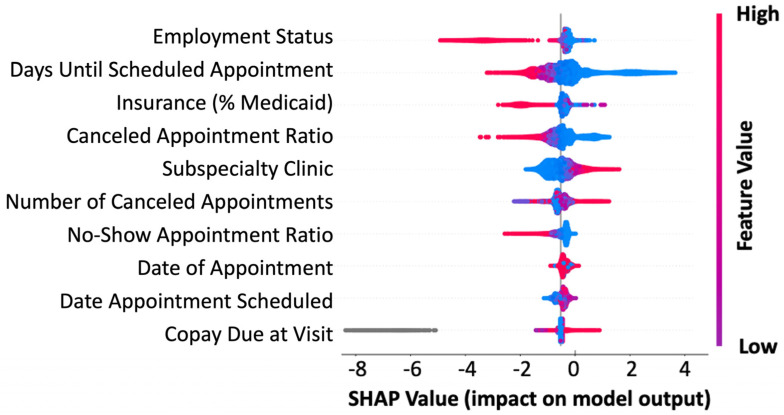
Feature importance in predicting no-shows. SHAP summary plot for the top 10 features contributing to the XGBoost model. Each line represents a feature, and the abscissa is the SHAP value. Red dots represent higher feature values, blue dots represent lower feature values, and gray dots represent missing values.

The machine learning model developed the no-show prediction probabilities. Table 2 highlights the superior prediction capacity for patient no-shows when using all 46 features collected (precision: 0.82, recall: 0.88) as well as the top 5 predictive features (precision: 0.81, recall: 0.84) in the validation dataset. This is further captured by the Receiver Operator Characteristic (ROC) area under the curve (AUC) for all features (AUC: 0.90) and the top five features (AUC: 0.88) (Figure 2). Additionally, we used the basic “no-show rate” alone to compute the likelihood of a patient being compliant with their visit (AUC: 0.64) (Figure 2). This “no-show rate” is a simple frequency: (total visits the patient has no-showed)/(total visits the patient has attended + total visits the patient has no-showed). This frequency is commonly employed by EMR systems to provide a baseline estimation if double booking or other alternative scheduling procedures should be enacted. To test if our algorithm can provide unbiased predictions across racial/ethnic groups, we subset the data. While Caucasians make up the primary patient population, our algorithm can efficiently generalize to other racial and ethnic populations, even when underrepresented (Table 3). From our analyses in the pediatric population, the no-show rate alone was insufficient to effectively predict patient compliance with their appointment. Additionally, features that were most important to the construction of the model were not within a single category, highlighting the complexity in interpretating patient no-shows (Figure 3). The most influential factors in predicting patient no-shows include employment status, appointment wait time, insurance type, cancellation history, and medical specialty (Table 4). Patients with full-time employment and shorter scheduling delays are more likely to attend, while those with Medicaid, frequent cancellations, and certain specialty appointments have higher no-show rates.

### 3.3. Evaluation of Model Generalizability and Adaptability to Unknown Healthcare Needs

**Figure 4:**
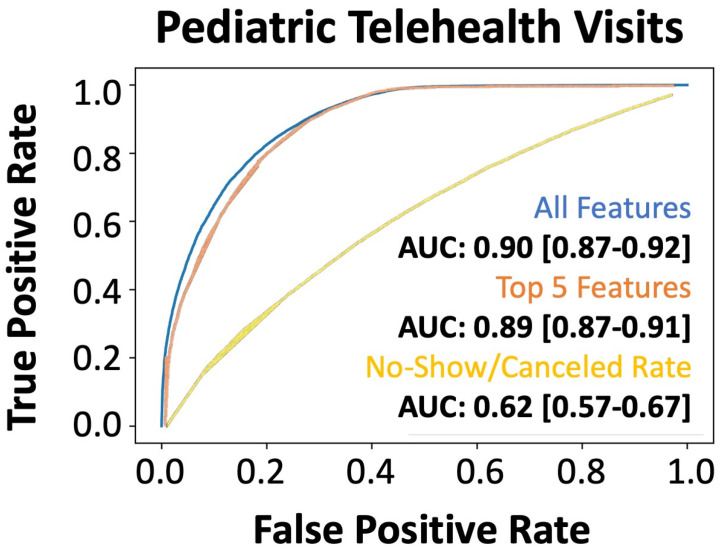
Performance of the machine learning (XGBoost) model for predicting no-shows in the external validation dataset. AUROC of xgboost machine learning model: all features (blue line), only the top five features (orange line), and the direct no-show/cancel rate (yellow line) in predicting no-shows in the 2020 telemedicine dataset during the pandemic.

We also wanted to understand if our model was adaptable to the changing dynamics of healthcare needs and initiatives, such as those driven by coronavirus disease 2019 (COVID-19), which created an increase in no-contact telehealth appointments. The evaluation of our holdout dataset (2020), which contained 35% telehealth visits, revealed that our model provided superior predictions across all features (AUC: 0.90) and the topmost predictive features (AUC: 0.88) (Figure 4). Again, we showed that the traditional “no-show rate” computed in the EMR system was significantly inferior to our integrative approach (AUC: 0.62). Model robustness on the 2020 pediatric telemedicine dataset and the shared top features between the validation and external holdout datasets highlight the persistence of our machine learning model in generating accurate predictions of patient no-shows. Additionally, the preliminary data are from pediatric appointments, highlighting our algorithm’s ability to predict the no-show rate of the patient based primarily on external factors, such as transportation by the guardian/caregiver.

For our system at WVU, we learned that strategic double booking might be a solution. Most of our clinics had 20 min visit slots, so for a 4 h clinic session, our proposed double booking was focused towards the middle of the session with limits of two per 4 h and three for 8 h. Some of our specialists had 30 min for a return visit and 1 h for a new patient visit. For those schedules, our strategy was to overbook a follow-up visit in the new patient visit slot with a new patient who had waited over 3 months for the appointment, as the data suggested decreased probability of patient adherence to a scheduled appointment after 3 months wait time. We strongly recommended not overbooking at the beginning or end of a clinic session to maintain the flow of the clinic as well as considering the providers’ efficiency during the clinic session. As technology improves, all these measures might even become second nature in clinic patient scheduling.

## 4. Discussion

Our technology uses an AI-based machine learning algorithm to predict the probability of individual patients not showing up to an appointment on a given date and time. The algorithm uses a patient’s historical demographic data to proactively predict no-shows and employ strategic double booking to avoid disruptions and minimize costs to the clinic. Unlike other scheduling systems that employ advanced statistical techniques (such as logistic regression and Bayesian prediction), our platform leverages cutting-edge machine learning technology—a scalable, distributed gradient-boosted decision tree algorithm utilizing minimal feature input for easy implementation across all healthcare systems—that continues to learn and improve, thus increasing prediction accuracy over time.

Our model maintained high predictive performance (AUC: 0.90) for telehealth appointments, which is important for generalization to hybrid appointment styles. Importantly, when examining the top five features used for no-show prediction, none of these features are specific to in-person visits. This further highlights that there are innate factors involved in appointment adherence that are separate from accessible transportation, weather conditions, and other factors contributing to an in-person visit. The marginal difference in performance between all features and the top five features (AUC: 0.90 vs. 0.88) can be explained by the types of included features. For example, important features, such as “canceled appointment ratio”, had multiple highly correlated features, including the number of changed appointments, number of canceled appointments, number of appointments attended, and no-show count. These highly correlated features added little value to the model performance. Further, during COVID-19, the increased use of telehealth altered patient behavior. Traditional factors such as travel distance became irrelevant, while new variables (e.g., internet connectivity, prior telehealth experience) gained importance. As discussed, features innate to the experience of telehealth or in-person visits were less contributory to model development overall, therefore allowing the model to generalize well between both populations.

Of the work that has been performed to improve patient no-show prediction, many studies have failed to perform superiorly to the basic statistic of no-show rate displayed per patient [17]. While the no-show rate metric (AUC: 0.64) is a commonly used heuristic, it lacks contextual awareness. It does not factor in temporal variations (e.g., seasonal trends), socioeconomic variables, or patient behavior, limiting its predictive capability compared to our machine learning model. Of the studies that performed better than the basic no-show rate statistic, most algorithms have not shown superior performance in predicting patient no-shows (i.e., AUC < 0.85) and have also only been applied to very specific populations (i.e., a single population or visit type) [17]. While the current algorithms predicting patient no-shows reveal promise for clinical application [23,24], there is currently no validated machine learning algorithm incorporated into an EMR to provide real-time predictions. Additionally, due to the limited scope of most no-show prediction algorithms, racial bias within the algorithm can result in up to a 30% increase in wait time for black patients [25]; optimizing care for patients, regardless of gender, race/ethnicity, and socioeconomic status, requires a demonstration of the algorithm’s stability across these conditions to promote healthcare equity.

Clinics currently utilize scheduling protocols that dictate how patients get scheduled for each clinic and within different specialties. There is considerable variability per provider, per clinic, and sometimes even per site within a healthcare system. Epic Systems Corporation (Epic) Electronic Medical Record (EMR) has a basic statistic that displays the no-show rate per patient in the scheduling software, but there is little knowledge about the prospective performance of this information. However, this information is not currently utilized in improving the scheduling of appointments.

While the scheduling staff can see the historic no-show rate for each patient when they call for an appointment, they have no autonomy to actively modify scheduling to reduce patient no-shows and follow the guidance of clinic protocols. As such, commonly implemented approaches to avoid no-shows include appointment reminders and no-show fines, while approaches to reduce the impact of no-shows on the providers and the healthcare system include double booking. Double booking could offer an advantage to both the patient and provider if implemented in a strategic manner, including through combination with predictive AI algorithms. However, such automated “strategic double booking” would require a machine learning algorithm capable of dynamically updating based on each patient’s likelihood of missing an appointment. For example, at WVU, strategic double booking was observed to be an effective approach. In clinics with 20 min visit slots, double booking was typically concentrated in the middle of 4 h sessions, with a common limit of two patients for a 4 h session and three for an 8 h session. For specialists with 30 min return visits and 1 h new patient appointments, follow-up visit slots were often overbooked with new patients who had been waiting over 3 months, as data suggested a decrease in adherence following such delays. Additionally, overbooking at the start or end of clinic sessions was generally avoided to maintain clinic flow and optimize provider efficiency.

To ensure operational feasibility, the algorithm must optimize computational efficiency by leveraging parallel processing and scalable infrastructure to handle large EMR datasets in real time. Data collection challenges, such as missing values and inconsistent formats across healthcare systems, require robust preprocessing pipelines with imputation and standardization techniques. Seamless integration with EMR systems demands API-based interoperability, ensuring secure, real-time data exchange without disrupting clinical workflows. Additionally, model updates should be automated to adapt to evolving patient behaviors and scheduling trends while maintaining interpretability for clinical decision making. We outline additional approaches for clinical application of the algorithm in Table 5.

## 5. Limitations

Our study looked at the clinic population in the Appalachian region of West Virginia. Even though the financial diversity within the state was taken into consideration, there might be factors that were not obvious in our results due to limited ethnic and racial diversity in the state of West Virginia. Further, although the model performed well across all groups, slight performance variability was observed, particularly in underrepresented populations. Future work could help offset class imbalance through oversampling techniques (e.g., the Synthetic Minority Over-sampling Technique (SMOTE) [26]) or fairness-aware techniques such as adversarial debiasing [27] to improve model robustness.

Another limitation is the lack of additional machine/deep learning models for comparison. For example, some novel approaches include genetic algorithms or hybrid genetic algorithms combined with standard machine learning approaches such as Gaussian naive Bayes, support vector machines, and XGBoost [28]. Genetic algorithms optimize feature selection and hyperparameters through evolutionary searching but are computationally expensive due to iterative stochastic sampling. XGBoost, a gradient boosting framework, builds decision trees sequentially, optimizing errors with parallelization and regularization for efficiency. While genetic algorithms explore a broader solution space, they lack the structured learning efficiency of XGBoost, which can be faster and more scalable.

While our study provides valuable insights, it is important to acknowledge limitations such as potential data biases and the retrospective nature of the analysis. Importantly, the data used in this study are provided by a specific hospital or hospital system, and thus, in pediatric populations the generalizability of the research results may be limited. Future research can extend the data source to other hospitals and to other population and age ranges to cross-validate our results or could focus on prospectively collecting data and implementing interventions to evaluate their effectiveness in reducing patient no-show rates.

Our proposed solution of strategic overbooking itself has its limitations. It depends on the protocols shared with the call center to offer certain slots for overbooking. We believe that a model actively analyzing the patient no-show data and proposing slots in real time using patient no-show history might be a better solution to take away any end user bias or human errors in interpreting the protocol implementation.

## 6. Conclusions

No-shows in healthcare settings pose significant challenges for both providers and patients. Understanding the underlying factors driving these no-shows is crucial for developing effective interventions to reduce their occurrence and enhance clinic efficiency. This study proposes the implementation of a machine learning framework to predict patient no-shows, offering hospitals a proactive approach to optimize their outpatient appointment systems. By accurately anticipating potential no-show behavior, healthcare facilities can implement targeted strategies such as personalized appointment reminders, flexible scheduling options, and provider-specific interventions to mitigate the impact of no-shows on healthcare delivery. These proactive measures not only improve clinic efficiency but also enhance patient satisfaction, provider productivity, and overall healthcare outcomes. Future research will explore dynamic scheduling systems that adjust patient appointments based on predictive no-show risk and investigate fairness-aware ML techniques to mitigate demographic biases.

## Figures and Tables

**Table 1:** Details of the study population and influence of the variables across the show and no-show appointments. “*” represents a Cohen Effect Size ≥ 0.1.

Parameter	Appointment Completed [95% CI] (N = 116,442)	Appointment Not Completed [95% CI] (N = 93,202)	*p*-Value	Cohen (Effect Size)
Patient’s Age (years)	11.56 [11.49–11.62]	11.27 [11.2–11.33]	<0.001	0.0267
Gender (% Male)	61,478 (52.80%)	48,409 (51.94%)	<0.001	0.0172
Race/Ethnicity (% Caucasian)	108,668 (93.32%)	86,103 (92.38%)	<0.001	0.0377
English Fluency (% Fluent)	114,644 (98.46%)	91,307 (97.97%)	<0.001	0.0397
Department				
Allergy/Immunology	12,811 (11.00%)	9643 (10.35%)	<0.001	0.021
Cardiology	18,532 (15.92%)	10,872 (11.66%)	<0.001	0.1162 *
Craniofacial Surgery	694 (0.60%)	564 (0.61%)	0.7879	0.0012
Cardiothoracic Surgery	1881 (1.62%)	1239 (1.33%)	<0.001	0.0227
Dermatology	1550 (1.33%)	1187 (1.27%)	0.2486	0.005
Endocrinology	12,739 (10.94%)	9290 (9.97%)	<0.001	0.0312
Gastroenterology	10,864 (9.33%)	11,661 (12.51%)	<0.001	0.1094 *
Hematology/Oncology	7350 (6.31%)	3342 (3.59%)	<0.001	0.1121 *
Infectious Diseases	1125 (0.97%)	920 (0.99%)	0.6276	0.0021
Neurology	11,847 (10.17%)	13,610 (14.60%)	<0.001	0.1465 *
Neonatology	1329 (1.14%)	1366 (1.47%)	<0.001	0.0305
Neurosurgery	4119 (3.54%)	3665 (3.93%)	<0.001	0.0214
Nephrology	6152 (5.28%)	6946 (7.45%)	<0.001	0.097
Orthopedics	13,942 (11.97%)	8209 (8.81%)	<0.001	0.0975
Plastic Surgery	304 (0.26%)	225 (0.24%)	0.3725	0.0039
Pulmonology	1812 (1.56%)	2425 (2.60%)	<0.001	0.0845
Sports Medicine	298 (0.26%)	153 (0.16%)	<0.001	0.0182
Surgery (General)	3071 (2.64%)	1823 (1.96%)	<0.001	0.0425
Urology	17,474 (15.01%)	19,321 (20.73%)	<0.001	0.1603 *
Other Departments	395 (0.34%)	351 (0.38%)	0.1533	0.0064
Days Until Scheduled Appointment	48.06 [47.68–48.44]	82.28 [81.74–82.82]	<0.001	0.5180 *
Appointment Length	25.05 [24.98–25.11]	26.45 [26.38–26.52]	<0.001	0.1251 *
Referral Required	16,837 (14.46%)	14,858 (15.94%)	<0.001	0.0421
Overbooked Time Slot	12,302 (10.56%)	5602 (6.01%)	<0.001	0.1482 *
Same Day Appointment	9926 (8.52%)	829 (0.89%)	<0.001	0.2734 *
Canceled Appointment Ratio	20.40% [20.29–20.51%]	29.07% [28.93–29.22%]	<0.001	0.4544 *
No-Show Appointment Ratio	3.65% [3.62–3.69%]	5.12% [5.07–5.17%]	<0.001	0.2249 *
Guardian Gender (% Male)	14325 (22.98%)	7380 (19.86%)	<0.001	0.0742
Employment Status				
Full-Time	58,784 (50.48%)	31,562 (33.86%)	<0.001	0.3324 *
Part-Time	1814 (1.56%)	4758 (5.11%)	<0.001	0.0669
Student	32,678 (28.06%)	977 (1.05%)	<0.001	0.0412
Unemployed	7903 (6.79%)	23,121 (24.81%)	<0.001	0.0725
Insurance (% Medicaid)	50,497 (43.37%)	35,233 (37.80%)	<0.001	0.1123 *
Copay Due at Visit	8.07 [7.98–8.16]	6.29 [6.2–6.38]	<0.001	0.1146 *
Maximum Temperature	65.23 [65.12–65.33]	63.94 [63.82–64.06]	<0.001	0.0699
Minimum Temperature	47.41 [47.31–47.51]	46.35 [46.24–46.47]	<0.001	0.0615
Temperature	56.19 [56.09–56.29]	55.03 [54.92–55.15]	<0.001	0.0673
Wind Chill	27.32 [27.21–27.43]	25.96 [25.83–26.09]	<0.001	0.1008 *
Precipitation	0.13 [0.13–0.13]	0.13 [0.13–0.13]	0.0235	0.01
Wind Speed	10.38 [10.36–10.4]	10.42 [10.4–10.44]	0.0057	0.0121
Cloud Cover	52.53 [52.35–52.72]	53.79 [53.59–54]	<0.001	0.0395
Relative Humidity	67.26 [67.19–67.34]	67.45 [67.37–67.53]	0.0011	0.0142
Weather—Clear	29,065 (24.96%)	22,124 (23.74%)	<0.001	0.0283
Weather—Rain	56,191 (48.26%)	45,990 (49.34%)	<0.001	0.0218
Weather—Overcast	35,816 (30.76%)	30,136 (32.33%)	<0.001	0.0341
Weather—Partially Cloudy	51,561 (44.28%)	40,942 (43.93%)	0.1066	0.0071

**Table 2:** Model performance metrics on the 2019 validation data.

	Precision	Recall	F1-Score
All Features			
Negative Class	0.83	0.75	0.79
Positive Class	0.82	0.88	0.85
Accuracy			0.82
Top 5 Features			
Negative Class	0.78	0.74	0.76
Positive Class	0.81	0.84	0.82
Accuracy			0.80

**Table 3:** Model training on the entire dataset followed by application and evaluation on individual racial and ethnic groups.

Racial/Ethnic Group	2015–2018 (# of Patients and Total Percent)	2019 (# of Patients and ROC AUC)	2020 (# of Patients and ROC AUC)
Caucasian	177,375 (93%)	67,559 (AUC: 0.89)	47,495 (AUC: 0.89)
Black	5644 (3.0%)	1999 (AUC: 0.84)	1466 (AUC: 0.83)
Two or More Races	4570 (2.4%)	1933 (AUC: 0.75)	1408 (AUC: 0.83)
Hispanic/Latino	2386 (1.2%)	1095 (AUC: 0.79)	731 (AUC: 0.81)
Asian American	1073 (0.6%)	568 (AUC: 0.83)	372 (AUC: 0.85)
Native American	204 (0.1%)	69 (AUC: 0.84)	84 (AUC: 0.78)

**Table 4:** Top five most predictive features based on SHAP analysis.

Rank	Feature	Feature Importance (F Score)	Influence on No-Shows
1	Employment Status	438	Full-time employment leads to fewer no-shows
2	Days Until Scheduled Appointment	105	Longer wait times increase no-show likelihood
3	Insurance (% Medicaid)	87	Socioeconomic status is a barrier to receiving care
4	Canceled Appointment Ratio	86	Higher previous cancellations correlate with no-shows
5	Department or Medical Specialty	52	Higher no-show rates in hematology, neurology, and gastroenterology

**Table 5:** Optimized double booking and EMR integration strategies.

Approach	Description
Optimized Double Booking	
Strategic Overbooking Optimization	Targets high-risk no-show patients for overbooking slots, pairing them with low-risk patients to reduce wasted provider time.
Risk-Based Appointment Reallocation	Dynamically reallocates appointments based on no-show risk, placing moderate-risk patients in flexible buffer slots.
Mixed Scheduling Approach	Distributes patient groups (high, moderate, low risk) throughout clinic schedules to balance efficiency and minimize provider downtime.
Integration with the EMR	
Automated No-Show Risk Score Generation	Generates no-show probabilities using patient history and real-time factors at the time of appointment booking.
Intelligent Scheduling Decision Engine	Integrates with EMR systems to suggest optimal scheduling decisions based on predicted no-show likelihood.
Adaptive Rebooking and Waitlist Management	Proactively engages high no-show risk patients with reminders or offers earlier slots to waitlist patients to maximize efficiency.

## Data Availability

All data produced in the present study are available upon reasonable request to the authors.
